# Stroke-Related Alterations in the Brain’s Functional Connectivity Response Associated with Upper Limb Multi-Joint Linkage Movement

**DOI:** 10.3390/brainsci13020338

**Published:** 2023-02-16

**Authors:** Qitong Chu, Xin Guo, Tengyu Zhang, Congcong Huo, Xuemin Zhang, Gongcheng Xu, Zhaoxin Lun, Shengcui Cheng, Ping Xie

**Affiliations:** 1 School of Artificial Intelligence and Data Science, Hebei University of Technology, Tianjin 300130, China; 2 Beijing Key Laboratory of Rehabilitation Technical Aids for Old-Age Disability, Key Laboratory of Neuro-Functional Information and Rehabilitation Engineering of the Ministry of Civil Affairs, National Research Center for Rehabilitation Technical Aids, Beijing 100176, China; 3 Engineering Research Center of Intelligent Rehabilitation Device and Detection Technology, Ministry of Education, Tianjin 300130, China; 4 Qinhuangdao Research Institute of National Research Center for Rehabilitation Technical Aids, Qinhuangdao 066004, China; 5 Beijing Advanced Innovation Centre for Biomedical Engineering, School of Biological Science and Medical Engineering, Beihang University, Beijing 100191, China; 6 Key Laboratory of Intelligent Rehabilitation and Neuromodulation of Hebei Province, School of Electrical Engineering, Yanshan University, Qinhuangdao 066004, China

**Keywords:** stroke, functional near infrared spectroscopy, functional connectivity, upper limb

## Abstract

Stroke is one of the primary causes of motor disorders, which can seriously affect the patient’s quality of life. However, the assessment of the upper limb affected by stroke is commonly based on scales, and the characteristics of brain reorganization induced by limb movement are not clear. Thus, this study aimed to investigate stroke-related cortical reorganization based on functional near infrared spectroscopy (fNIRS) during upper limb multi-joint linkage movement with reference to the Fugl–Meyer Assessment of the upper extremities (FMA-UE). In total, 15 stroke patients and 15 healthy subjects participated in this study. The functional connectivity (FC) between channels and the regions of interest (ROI) was calculated by Pearson’s correlation coefficient. The results showed that compared with the control group, the FC between the prefrontal cortex and the motor cortex was significantly increased in the resting state and the affected upper limb’s multi-joint linkage movements, while the FC between the motor cortex was significantly decreased during the unaffected upper limb’s multi-joint linkage movements. Moreover, the significantly increased ROI FC in the resting state showed a significantly positive correlation with FMA-UE in stroke patients (*p* < 0.05). This study highlights a new biomarker for evaluating the function of movement in stroke patients and provides guidance for rehabilitation training.

## 1. Introduction

According to statistics from the World Health Organization, stroke has become one of the major causes of human death and disability worldwide [1]. There are approximately 2.5 million new cases of stroke and 1.5 million deaths every year in China. Poststroke motor dysfunction is a common complication that mainly manifests as insufficient muscle strength, abnormal muscle tone and abnormal postural reflex, which reduce the stability of body movements, and the precision and coordination of motor functions [2]. The upper limbs are among the most important and most used parts of the human motor system. Most activities need to be completed by the upper limbs in daily life. However, functional recovery of the upper limbs after stroke is difficult. Clinical assessments of stroke-related motor disorders mainly rely on scales such as the Fugl–Meyer Assessment (FMA), and the pattern of cortical reorganization is not clear. Therefore, it is necessary to explore the stroke-related brain recombination patterns associated with upper limb dyskinesia.

The development of medical imaging technology has provided a new approach to assessing the motor functions after stroke at the neurophysiological level [3]. However, these traditional functional brain imaging techniques have corresponding limitations. For example, although functional magnetic resonance imaging (fMRI) has strong spatial resolution [4], it has low temporal resolution, poor convenience and a high diagnostic cost. Electroencephalograms (EEG) have strong temporal resolution and have the advantages of being simple and rapid, but the spatial resolution of EEG is low [5,6,7]. Functional near infrared spectroscopy (fNIRS), a noninvasive optical imaging technique, can be applied at the tissue surface through an optical probe [8,9]. Changes in the concentrations of oxyhemoglobin (delta HbO_2_) and deoxyhemoglobin (delta HbR) can be measured by emitting and receiving near-infrared light (650–1000 nm) to reflect the hemodynamic changes in the cerebral tissues [10,11,12,13,14], providing a noninvasive, real-time, dynamic and repeatable method of detecting brain function for stroke patients.

Recently, fNIRS has been widely used in the field of stroke rehabilitation [15,16,17,18]. Huo et al. [19] applied fNIRS techniques to measure the cortical response induced by specific motor training paradigms in stroke patients. The results showed that obvious cortical activation was observed in the bilateral hemispheres during task-oriented training for the upper limb. Liang et al. [20] found that the cortical activation measured by fNIRS was associated with ankle dorsiflexion tasks in stroke patients and provided a fNIRS-based biomarker for the recovery of motor function. Arun et al. [21] found that functional connectivity (FC) of the motor cortex of stroke patients in the resting state gradually increased. Delorme et al. [22] found that there was a shift from bilateral activation to unilateral activation when performing simple unilateral upper limb motor tasks during motor recovery after a stroke.

Most of the abovementioned studies focused on the changes in resting-state FC and brain activation when performing motor tasks in stroke patients, and there are few studies on FC during specific upper limb tasks. The purpose of this study was to investigate the cerebral cortical reorganization patterns by applying fNIRS during multi-joint linkage movements of the upper limb in stroke patients. We hypothesized that the characteristics of cortical network reorganization could be identified by fNIRS during multi-joint linkage movements of the upper limb in stroke patients. This study might help us to understand the pattern of cortical reorganization during specific upper limb movements and provide personalized rehabilitation treatment plans for stroke patients.

## 2. Materials and Methods

### 2.1. Participants

In total, 30 participants, including 15 stroke patients and 15 age-matched healthy individuals without neurological or psychiatric diseases (control group), participated in this study. One healthy participant was excluded due to the excessive motion artifacts. Therefore, 15 patients and 14 healthy subjects were finally used for the analysis in this study. All participants in the patient group prior to experiencing a stroke and those in the control group were right-handed. The handedness was determined by self-report and confirmed by the Chinese edition of the Handedness Inventory [23]. For stroke patients, the inclusion criteria were as follows: (1) 2 weeks from the onset of stroke, (2) unilateral limb motor dysfunction, and (3) normal cognition and no impairment in verbal communication. The exclusion criteria were as follows: (1) any clinically significant or unstable medical disorder, (2) any neuropsychiatric comorbidity other than stroke and (3) a metal implant in the brain. The clinical characteristics of the stroke patients are shown in Table 1.

### 2.2. Experimental Design and fNIRS Recordings

The multi-joint linkage movement of the upper limb was designed on the basis of the joints involved in the FMA-UE, which assesses single-joint and multi-joint movements, out-of-synergy movements, digit individuation, movement speed, dysmetria, ataxia and reflexes [24,25]. The multi-joint linkage movement of the upper limb included touching the back of one hand to the same ear then holding the position (Task 1) and touching one hand to the opposite knee then holding the position (Task 2). Specifically, Task 1 was designed on the basis of the flexor synergy of volitional movement within the synergy of FMA-UE, including shoulder retraction, shoulder elevation, shoulder abduction, external rotation of the shoulder, elbow flexion, forearm supination and wrist flexion. Task 2 was designed on the basis of the extensor synergy of volitional movement within the synergy of FMA-UE, including shoulder adduction, internal rotation of the shoulder, elbow extension, forearm pronation and wrist extension. The shoulder, elbow and wrist joints were included in both tasks.

**Figure 1:**
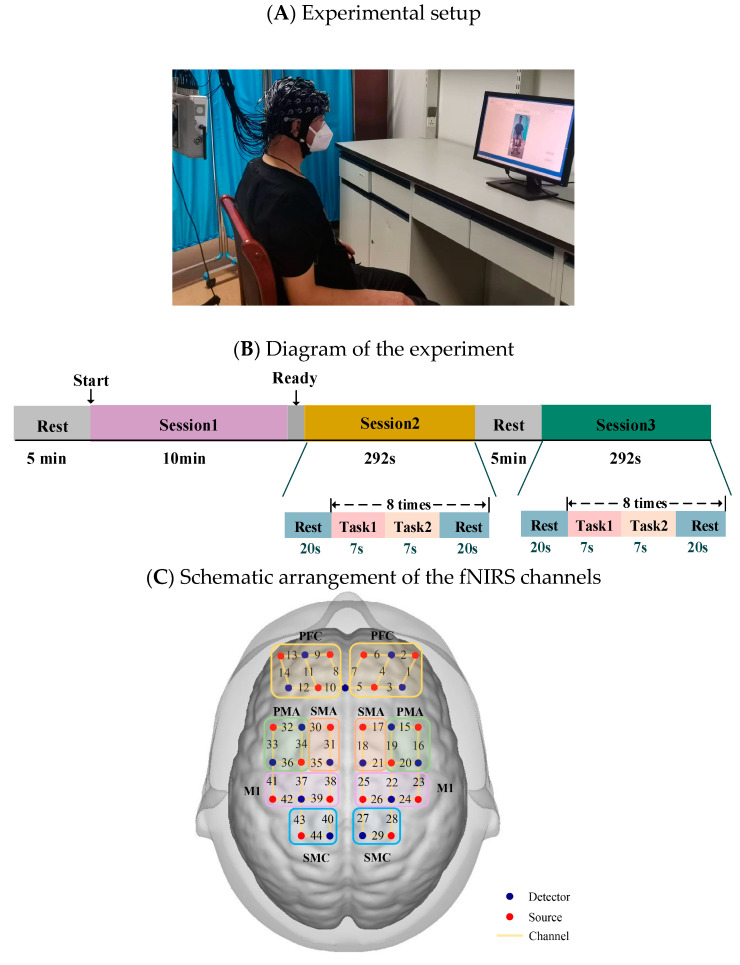
(**A**) Experimental setup. (**B**) Diagram of the experiment. Session 1: resting state; Session 2: multi-joint movement of the upper limb on the unaffected side (or the dominant side in healthy control individuals); Session 3: multi-joint movement of the upper limb on the affected side (or the nondominant side in healthy control individuals). (**C**) Schematic arrangement of the fNIRS channels. The red dots represent detectors, the blue dots represent sources, and channels are represented by yellow lines with the corresponding numbers. fNIRS, functional near infrared spectroscopy; PFC, prefrontal cortex; SMA, plementary motor area; PMA, premotor area.

All participants were seated comfortably in front of a computer screen in a quiet, dimly lit room. They were first instructed to relax for 5 min to calm down. They were then instructed to maintain a comfortable sitting position and complete the following 3 sessions by following the text instructions on the screen: resting state (Session 1), multi-joint linkage movement of the unaffected upper limb or the dominant side in healthy control individuals (Session 2), and multi-joint linkage movement of the affected upper limb or the nondominant side in healthy control individuals (Session 3), as shown in Figure 1A. The experimental guidance interface was designed on the MATLAB GUI, which strictly controlled the time according to the experimental design. During the three sessions of the experiment, the corresponding text and picture were displayed on the screen according to the experimental task. In the resting state session, they were asked to relax with their eyes closed but stay awake for 10 min. Next, the unilateral upper limb multi-joint linkage movement was designed as a block experiment. Specifically, each trial consisted of a 14-s task and 20 s of rest, as shown in Figure 1B. In the 14-s task, the participants were asked to touch their ear with the back of the hand for the first 4 s and maintaining the contact for 3 s, and then touching the opposite knee with the palm of the hand and maintaining the contact for 3 s Participants first performed 8 trials using the unaffected upper limb (or the dominant side in healthy control individuals) and then performed 8 trials using the affected upper limb (or the nondominant side in healthy control individuals). Relaxing for at least 5 min between Sessions 2 and 3 was performed to avoid the effects of the previous session.

The fNIRS signals were acquired using a multichannel commercial fNIRS system (NirSmart, HuiChuang, China). The sampling rate was 11 Hz, and the wavelengths used were 730 nm and 850 nm. The probes (18 sources and 15 detectors) were placed to cover the prefrontal cortex and movement-related cortex according to the international 10–10 system. The fNIRS channels were divided into 10 regions of interest (ROIs) distributed in the bilateral hemisphere: the prefrontal cortex (PFC), the premotor area (PMA), the supplementary motor area (SMA), the primary motor cortex (M1) and the sensorimotor cortex (SMC), as shown in Figure 1C.

### 2.3. Data Analysis

#### 2.3.1. Data Preprocessing

For ease of analysis, the left and right brain hemispheres of patients with right hemiplegia were symmetrically transformed; specifically, the right side was equivalent to the ipsilesional hemisphere, and the left side was equivalent to the contralesional hemisphere in all patients.

Data analysis and processing in this research were based on MATLAB 2021b. First, the channels with oversaturated light intensity recorded during the experiment were deleted manually, the original light intensity of each channel in each data point was checked, and channels that were not in the range of 0.5–1000 dB were eliminated. Next, the raw light intensity signals acquired by NirSmart were converted into optical density signals. To correct the motion artifacts, the moving standard deviation method was used to detect motion artifacts in the data, the standard deviation threshold was set at 6, and the peak threshold was set at 0.5. The motion artifacts were removed by cubic spline interpolation using the csaps function in MATLAB. The peak noise in the data was removed by the moving average. The corrected signals were then filtered by a 0.01–0.2 Hz bandpass filter to remove physiological noise caused by heartbeats, respiration and machine noise [21,26]. Finally, the optical density signal was converted into the delta HbO_2_ and delta HbR by the modified Beer—Lambert law [27]. Because of the greater signal-to-noise ratio of the delta HbO_2_ signal and less contamination of the delta HbR signal by global processes, we used the delta HbO_2_ and delta HbR signals in the remaining analyses. The DPF was set as 6 for both the wavelengths used [28], and the formula was as follows:

(1) $$\left[\begin{array}{c} \Delta [HHb]\\ \Delta [HbO_{2}] \end{array}\right]={\left(d\right)}^{-1}{\left[\begin{array}{cc} \varepsilon_{HHb,\lambda_{1}} & \varepsilon_{HbO_{2},\lambda_{2}}\\ \varepsilon_{HHb,\lambda_{2}} & \varepsilon_{HbO_{2},\lambda_{2}} \end{array}\right]}^{-1}\left[\begin{array}{c} \Delta OD(\Delta t,\lambda_{1})/DPF(\lambda_{1})\\ \Delta OD(\Delta t,\lambda_{2})/DPF(\lambda_{2}) \end{array}\right]$$

#### 2.3.2. FC Analysis

For each subject, the Pearson correlation coefficient was used to calculate the correlation between each pair of channels, and the Pearson correlation coefficient was formulated as follows:

(2) $$r=\frac{\sum_{i=1}^{n}\left(X_{i}-\bar{X})(Y_{i}-\bar{Y}\right)}{\sqrt{\sum_{i=1}^{n}{\left(X_{i}-\bar{X}\right)}^{2}}\sqrt{\sum_{i=1}^{n}{\left(Y_{i}-\bar{Y}\right)}^{2}}}$$

After the Pearson correlation coefficient between the two channels had been calculated, the results were standardized by Fisher’s z, which can correct the nonlinearity of the Pearson correlation coefficient. The FC matrix between the channels calculated by each subject had 44 × 44 dimensions. The formula was as follows:

(3) $$z=\frac{1}{2}\ln\left(\frac{1+r}{1-r}\right)=ar\tanh(r)$$

Because of the low spatial resolution of fNIRS, the channel-wise FC matrix was averaged over the ROIs according to the distribution of the fNIRS channels, generating a 10 × 10 FC matrix of the ROIs, which was then statistically analyzed.

#### 2.3.3. Statistical Analysis

Statistical analysis was performed for the channel-wise and ROI-wise FCs within groups and between groups. First, a paired sample t-test was conducted for the FC between the left upper limb’s multi-joint linkage and the resting state in the control group. In the stroke patient group, a paired sample *t*-test was conducted for the FC between the unaffected upper limb’s multi-joint linkage movement and the resting state, and between the affected upper limb’s multi-joint linkage movement and the resting state. Via Bonferroni’s correction method, the paired sample *t*-test was compared three times within the group, so the significance level was set to *p* < 0.05/3, which is *p* < 0.017.

Next, for the comparison between the groups, an independent sample t-test was conducted on the channel-wise FC in the resting state between the patient group and the control group, between multi-joint linkage movement of the upper limb on the dominant side in healthy controls and multi-joint linkage movement of the unaffected upper limb in the patient group, and between multi-joint linkage movement of the upper limb on the nondominant in healthy controls and multi-joint linkage movement of the affected upper limb of the patient group. Bonferroni’s correction method was used because the independent sample t-test between the groups was compared three times, and the significance level was set to *p* < 0.05/3, which is *p* < 0.017.

## 3. Results

### 3.1. Task-Related Changes in the FC Based on Delta HbO_2_ in the Control Group

**Figure 2:**
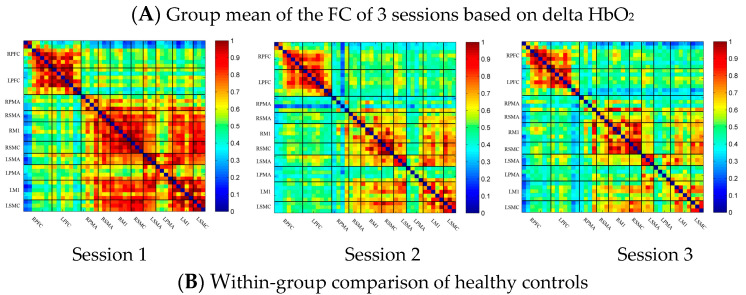

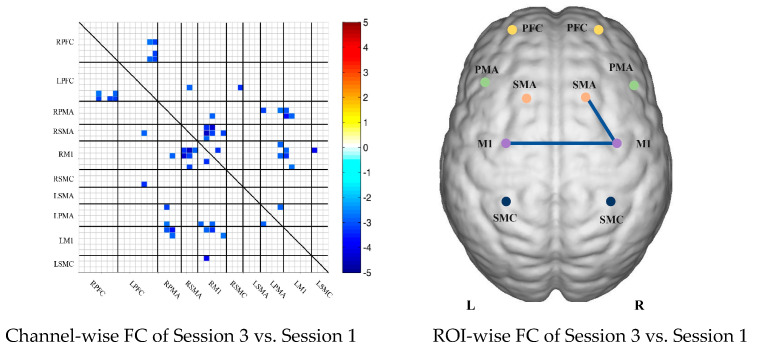
(**A**) Mean FC over 3 sessions in the healthy control group based on delta HbO_2_. Session 1: resting state; Session 2: multi-joint linkage movement of the dominant (right) upper limb; Session 3: multi-joint linkage movement of the nondominant (left) upper limb. (**B**) Within-group comparison of healthy controls. Blue indicates that the FC of Session 2 was significantly lower than that of Session 1. HbO_2_, oxyhemoglobin; RPFC, right prefrontal cortex; LPFC, left prefrontal cortex; RPMA, right premotor area; LPMA, left premotor area; RSMA, right supplementary motor area; LSMA, left supplementary motor area; RM1, right primary motor cortex; LM1, left primary motor cortex; RSMC, right sensorimotor cortex; LSMC, left sensorimotor cortex.

In the control group, the channel-wise FC based on delta HbO_2_ was arranged according to the sequence of ROIs during the resting state, multi-joint linkage movement of the dominant upper limb and multi-joint linkage movement of the nondominant upper limb, as shown in Figure 2A. As shown in the figure, the resting state’s FC was significantly stronger than that in the task-induced state.

Compared with the resting state, the FC during multi-joint linkage movement of the nondominant upper limb decreased significantly, and was mainly distributed in the homologous prefrontal cortex and motor cortex, and within the right motor cortex. The channel pairs with significant differences were arranged according to the order of the divided ROIs, as shown in Figure 2B. In the ROI-wise analysis, the intensity of the FC induced by movement of the left upper limb was significantly lower than that in the resting state, namely in RSMA-RM1 and RM1-LM1, as shown in Figure 2B.

### 3.2. Task-Related Changes in the FC Based on Delta HbR in the Control Group

**Figure 3:**
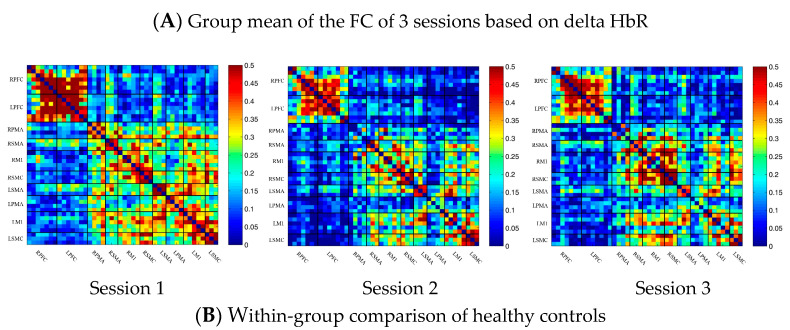

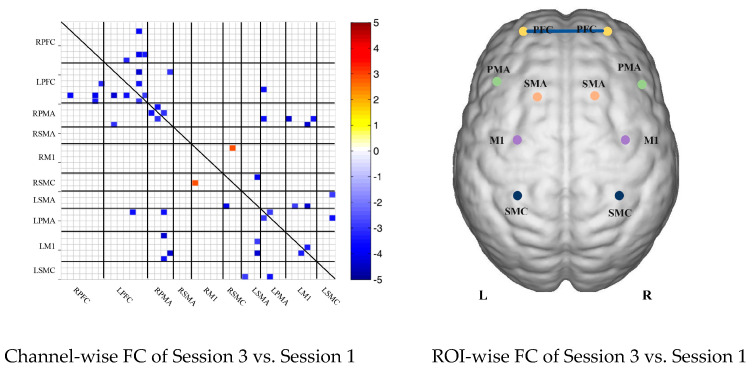
(**A**) Mean FC of 3 sessions in the healthy control group based on delta HbR. Session 1: resting state; Session 2: multi-joint linkage movement of the dominant (right) upper limb; Session 3: multi-joint linkage movement of the nondominant (left) upper limb. (**B**) Within-group comparison of healthy controls. Blue indicates that the FC of Session 2 was significantly lower than Session 1. HbR, deoxyhemoglobin.

In the control group, the channel-wise FC based on delta HbR was arranged according to the sequence of ROIs during the resting state, multi-joint linkage movement of the dominant upper limb and multi-joint linkage movement of the nondominant upper limb, as shown in Figure 3A. As in the results of channel-wise FC based on delta HbO_2_, the resting state FC was significantly stronger than that in the task-induced state.

Compared with the resting state, the FC during multi-joint linkage movement of the upper limb on the nondominant side decreased significantly, mainly in the bilateral prefrontal cortex, and a small amount in the bilateral motor cortex. The channel pairs with significant differences were arranged according to the order of the divided ROIs, as shown in Figure 3B. In the ROI-wise analysis, the intensity of the FC induced by movement of the left upper limb was significantly lower than that in the resting state, namely in RPFC-LPFC, as shown in Figure 3B.

### 3.3. Task-Related Changes in the FC Based on Delta HbO_2_ in the Stroke Group

**Figure 4:**
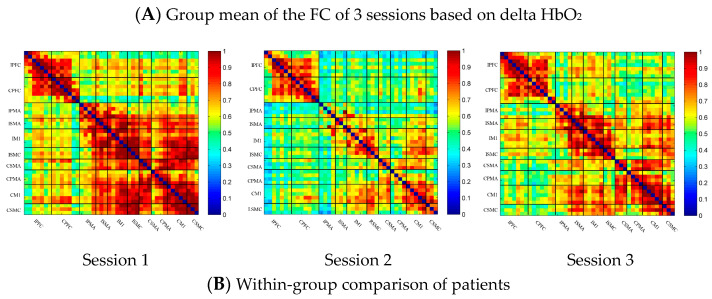

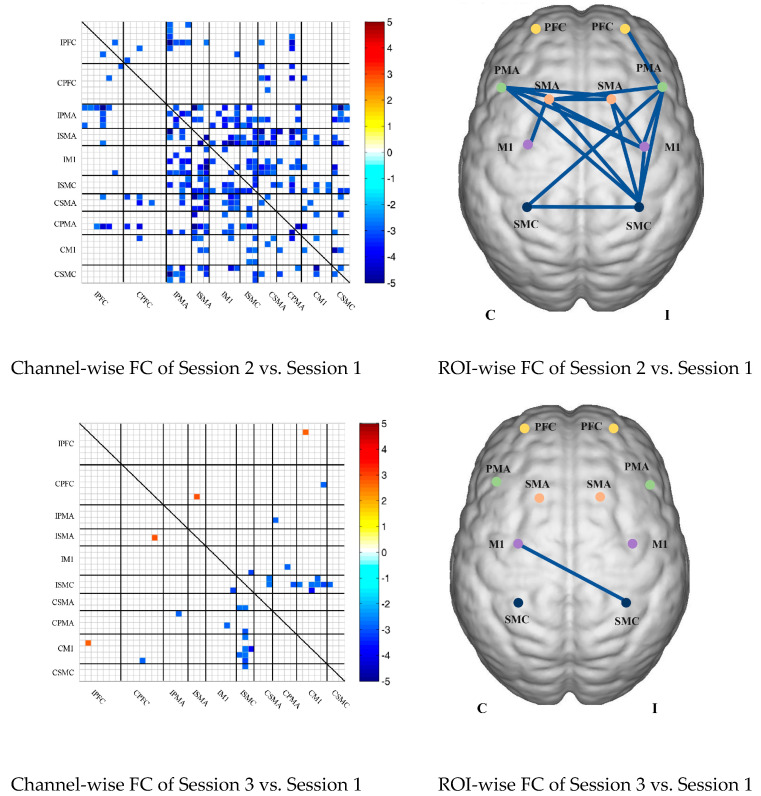
(**A**) Mean FC of 3 sessions in the stroke patient group based on delta HbO_2_. Session 1: resting state; Session 2: multi-joint linkage movement of the unaffected upper limb; Session 3: multi-joint linkage movement of the affected upper limb. (**B**) Within-group comparison of stroke patients. Blue indicates that the FC of Session 2/3 was significantly lower than that of Session 1, red indicates that the FC of Session 2/3 was significantly stronger than that of Session 1. CPFC, contralesional prefrontal cortex; IPFC, ipsilesional prefrontal cortex; CPMA, contralesional premotor area; IPMA, ipsilesional premotor area; CSMA, contralesional supplementary motor area; ISMA, ipsilesional supplementary motor area; CM1, contralesional primary motor cortex; IM1, ipsilesional primary motor cortex; CSMC, contralesional sensorimotor cortex; ISMC, ipsilesional sensorimotor cortex.

In the patient group, the channel-wise FC of each subject was arranged according to the sequence of ROIs during the resting state, multi-joint linkage movement of the unaffected upper limb and multi-joint linkage movement of the affected upper limb, as shown in Figure 4A. The resting state’s FC was significantly stronger than the task-induced FC, but the FC during movement of the affected upper limb in the patient group was stronger than that in the resting state in the prefrontal cortex.

Compared with the resting state, the channel-wise FC was significantly reduced during multi-joint linkage movement of the unaffected upper limb in the patients and was mainly distributed in the ipsilesional prefrontal cortex and ipsilesional motor cortex, as shown in Figure 4B. During multi-joint linkage movement of the affected upper limb, the channel-wise FC was significantly reduced and was mainly distributed in the ipsilesional motor cortex, and a few channel-wise FCs in the bilateral prefrontal cortex were significantly enhanced, as shown in Figure 4B.

In the ROI-wise FC, compared with the resting state, the FC of multi-joint linkage movement of the unaffected upper limb decreased significantly in the following ROIs: IPFC-IPMA, IPMA-IM1, IPMA-ISMC, IPMA-CSMA, IPMA-CSMC, ISMA-IM1, ISMA-ISMC, ISMA-CSMA, ISMA-CPMA, IM1-ISMC, IM1-CSMA, IM1-CPMA, ISMC-CSMA, ISMC-CPMA, ISMC-CSMC, CSMA-CPMA and CSMA-CM1, as shown in Figure 4B. During multi-joint linkage movement of the affected upper limb, the FC decreased significantly in the LM1-RSMC ROI, as shown in Figure 4B.

### 3.4. Task-Related Changes in the FC Based on Delta HbR in the Stroke Group

**Figure 5:**
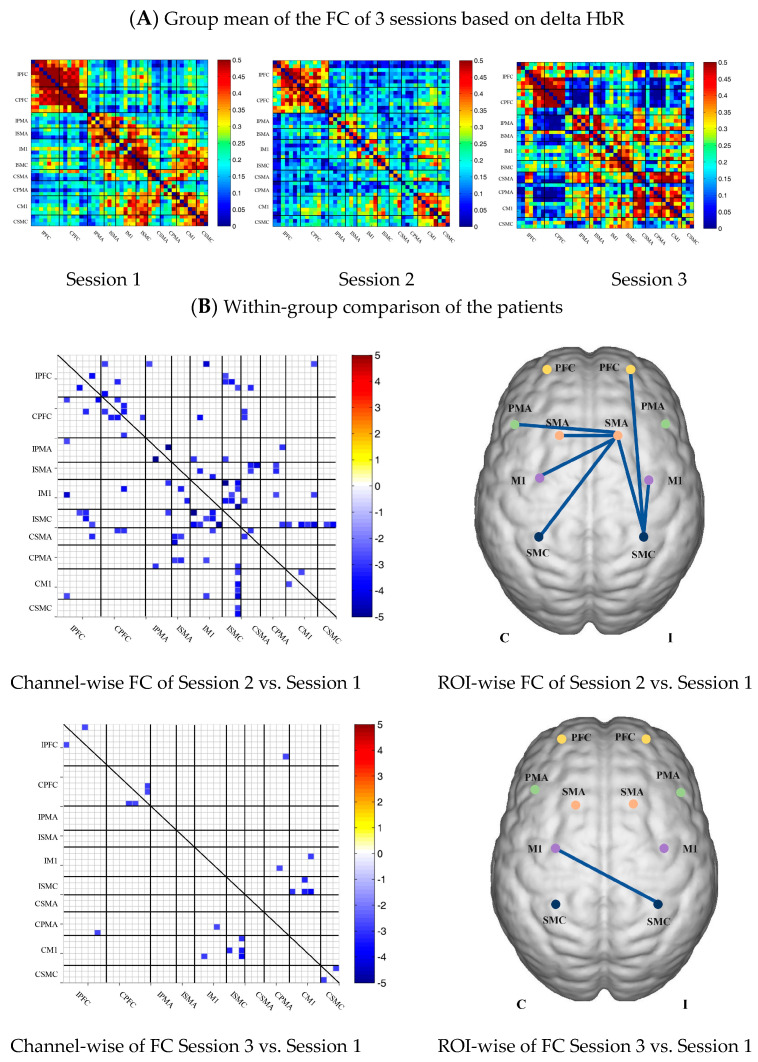
(**A**) Mean FC of 3 sessions in the stroke patient group based on delta HbR. Session 1: resting state; Session 2: multi-joint linkage movement of the unaffected upper limb; Session 3: multi-joint linkage movement of the affected upper limb. (**B**) Within-group comparison of stroke patients. Blue indicates that the FC of Session 2/3 was significantly lower than that of Session 1; red indicates that the FC of Session 2/3 was significantly stronger than that of Session 1.

In the patient group, the channel-wise FC based on HbR in each subject was arranged according to the sequence of ROIs during the resting state, multi-joint linkage movement of unaffected upper limb and multi-joint linkage movement of the affected upper limb, as shown in Figure 5A.

Compared with the resting state, the channel-wise FC based on HbR was significantly reduced during multi-joint linkage movement of the unaffected upper limb in the patients, and was mainly distributed in the ipsilesional prefrontal cortex, ipsilesional motor cortex and bilateral motor cortex, as shown in Figure 5B. During multi-joint linkage movement the affected upper limb, the channel-wise FC was significantly reduced and mainly distributed in the ipsilesional motor cortex, as shown in Figure 5B.

In the ROI-wise FC, compared with the resting state, the FC of the multi-joint linkage movement of the unaffected upper limb decreased significantly in the following ROIs: IPFC-ISMC, ISMA-IM1, ISMA-ISMC, ISMA-CSMA, ISMA-CPMA, ISMA-CSMC, IM1-ISMC, ISMC-CM1. During multi-joint linkage movement of the affected upper limb, the FC decreased significantly in the CM1-ISMC ROI, as shown in Figure 5B.

### 3.5. Comparison of the FC Based on Delta HbO_2_ between the Control and Patient Groups

**Figure 6:**
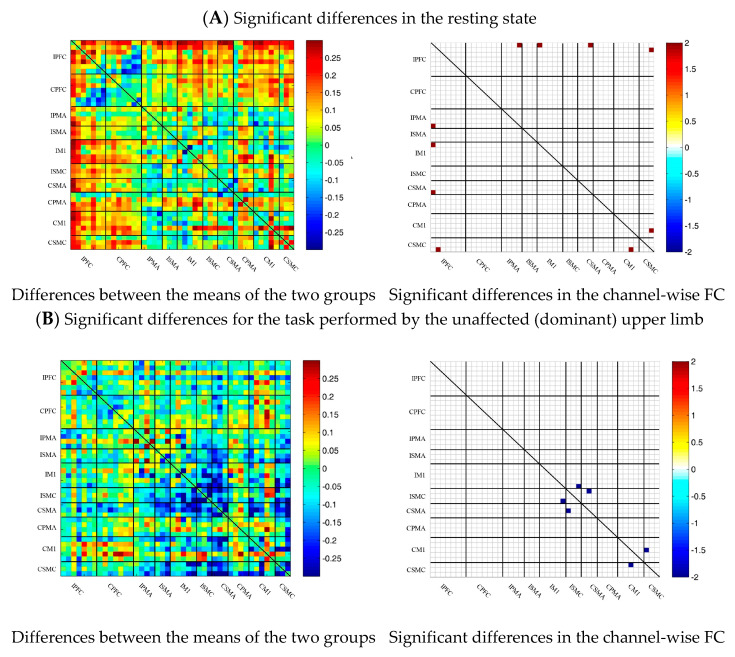

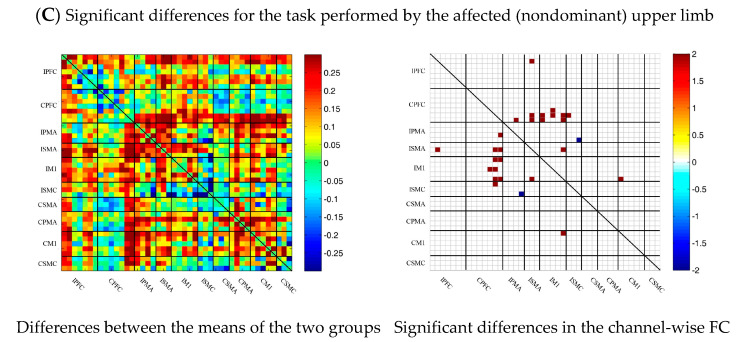
Comparison of the FC based on delta HbO_2_ between the patient and control group in different sessions. (**A**) Resting state; (**B**) multi-joint linkage movement of the unaffected (dominant) upper limb; (**C**) multi-joint linkage movement of the affected (nondominant) upper limb. Blue indicates that the FC of the patient group was lower than that of the control group; red indicates that the FC of the patient group was stronger than that of the control group.

To describe the differences between the patient group and the control group more clearly, the differences between the two groups were drawn first. As shown in Figure 6A, the FC between the bilateral prefrontal cortex and the motor cortex of the patient group was stronger than that of the control group, while the FC between the motor cortex of the patient group was generally lower than that of the control group. After statistical analysis, channels with significant differences were marked, namely 20-1, 22-1, 35-1 and 44-41, as shown in Figure 6A.

During multi-joint linkage movement of the unaffected (or dominant) upper limb, the difference between the average FC of the patient group and that of the control group was used to represent the difference between the two groups. In the whole brain, the FC of multi-joint linkage movement of the unaffected upper limb in the patient group was lower than that of the control group, as shown in Figure 6B. After statistical analysis, it was found that the channels with significant differences were as follows: 29-26, 31-27 and 40-39. These channels are mainly distributed in the motor cortex, as shown in Figure 6B.

During multi-joint linkage movement of the affected (or nondominant in the control group) upper limb, the difference between the average FC of the patient group and that of the control group was used to represent the difference between the two groups. The FC between the bilateral prefrontal cortex and the ipsilesional motor cortex of the patient group was higher than that of the control group, as shown in Figure 6C. After statistical analysis, the channels with significant differences were 18-2, 18-13, 18-14, 19-14, 22-13, 22-14, 24-12, 24-13, 25-13, 25-14, 25-14, 25-18, 27-13, 29-20 and 37-26, and the FC of the 29-20 channel pair was significantly lower in the patient group than in the healthy group. For the other channel pairs, the FC of the patient group was larger than that of the healthy group, as shown in Figure 6C.

### 3.6. Comparison of the FC Based on Delta HbR between Control and Patient Groups

**Figure 7:**
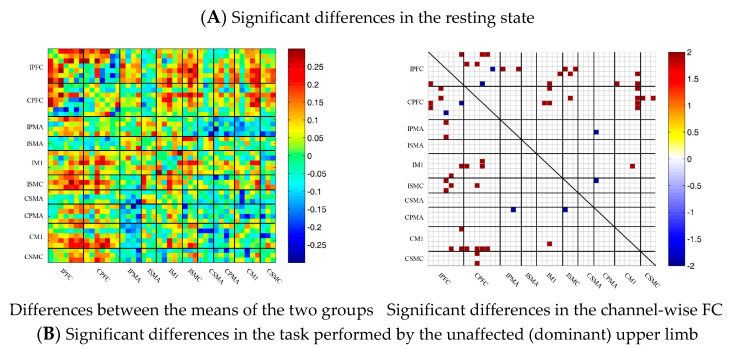

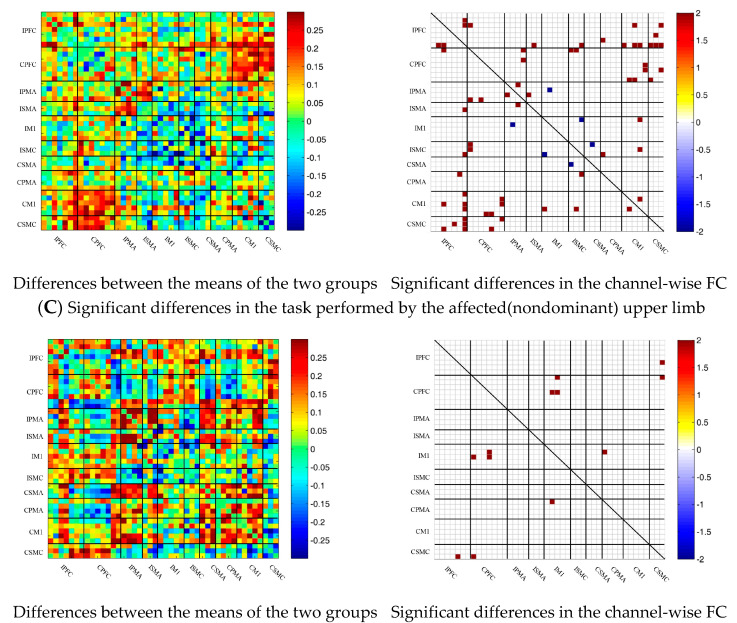
Comparison of the FC based on delta HbR between the patient and control groups in different sessions. (**A**) Resting state; (**B**) multi-joint linkage movement of the unaffected (dominant) upper limb; (**C**) multi-joint linkage movement of the affected (nondominant) upper limb. Blue indicates that the FC of the patient group was lower than that of the control group; red indicates that the FC of the patient group was stronger than that of the control group.

Similar to the method of analysis based on delta HbO_2_, the results of the comparison of the FC based on delta HbR between the control and patient groups are shown as Figure 7. During the resting state and multi-joint linkage movement of the affected (or nondominant in the control group) upper limb, the results of comparisons of the FC based on delta HbR between the two groups were consistent with those of delta HbO_2_. During multi-joint linkage movement of the unaffected (or dominant) upper limb, the FC of a few channel pairs distributed in motor cortex was significantly lower in the patient group than in the healthy group. The difference was that the FC between the motor cortex and the prefrontal cortex was stronger in the patient group than in the healthy group.

### 3.7. Correlation Analysis between the ROI-Wise FC and FMA-UE

**Figure 8:**
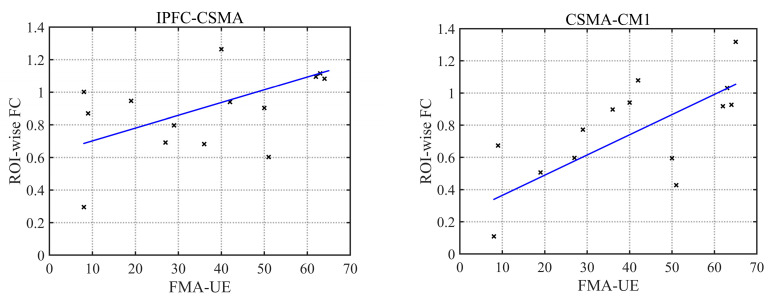
Correlation analysis of FC and FMA-UE.

A correlation analysis between the ROI-wise FC and movement of the upper limb on the affected side in the patient group showed that the FC of the IPFC-CSMA ROI in the resting state was positively correlated with the FMA-UE score of the affected upper limb (r = 0.59, *p* = 0.02), while during multi-joint linkage movement the unaffected upper limb, the FC of the CSMA-CM1 ROI was positively correlated with the FMA-UE of the affected upper limb (r = 0.73, *p* = 0.002), as shown in Figure 8.

## 4. Discussion

In this study, we applied FC in combination with fNIRS to investigate stroke-related cortical reorganization. Crucially, we analyzed the FC based on delta HbO_2_ and HbR during upper limb multi-joint linkage movement. There were three main findings. First, in both the control group and the patient group, the task-related FC was significantly lower than the resting state’s FC in the analysis of ROIs. Second, in the resting state, the FC between the ipsilesional prefrontal cortex and the bilateral motor cortex was significantly higher in the patient group than in the control group, and the FC in the ipsilesional prefrontal cortex and the contralesional motor cortex was significantly and positively correlated with the FMA-UE score. Third, in the patient group, only the ROIs on the contralesional side were in high synchronization during the multi-joint linkage movement of the unaffected upper limb. During multi-joint linkage movement the affected upper limb, the FC between the contralesional prefrontal cortex and the ipsilesional motor cortex was significantly higher than that of the control group.

Contrary to our common perceptions, the FC is not similar to the activation value; resting state activation is small, but dynamic activation is strong. First, FC refers to the mutual relationship between various cerebral cortex regions involved in neurophysiological activities. In the resting state, there is no external stimulus or induction of motor tasks, and spontaneous signals in the brain are not random noises but exist in a specific way [29], and they can be used to study the functional level of the whole brain [30,31]. Second, from the perspective of energy metabolism, the weight of the human brain only accounts for 2% of the total weight of the human body, but the resting state consumes 20% of the energy, most of which is used to maintain the transmission of nerve signals in the brain. When an exercise task is triggered, the brain usually uses only a small amount of energy, approximately 5%. This is consistent with our research results, and the results of the delta HbO_2_ and delta HbR tests together indicated that the strength of the FC in the resting state was greater than that induced by the motor task.

A stroke alters the brain’s resting state FC [32]. Previous studies have shown that the FC between the lesion and the ipsilateral prefrontal cortex is significantly enhanced in patients after a stroke [33]. In this study, compared with the control group, the patient group produced stronger FCs, and these significantly enhanced channels mainly existed between the ipsilesional prefrontal cortex and bilateral motor cortex. This may be related to network reorganization of the affected prefrontal cortex and bilateral motor cortex. The sensorimotor cortex and supplementary motor area of the contralesional side usually have the ability to maintain body balance and control body stability [34]. The ipsilesional motor cortex of the patients was damaged, and the balancing ability of the patients was worse than that of the control group. In the resting state, greater effort is needed to maintain the balance of the body and control the limbs in a static state, thus forming a compensatory pathway between the ipsilesional prefrontal cortex and the contralesional motor cortex. Moreover, the FC of the IPFC-CSMA was positively correlated with the FMA-UE score. When the motor function recovery of patients was better, the FC of IPFC-CSMA was stronger and a more complete compensatory pathway was established. Therefore, not only is the FC between the ipsilesional prefrontal cortex and the contralesional motor cortex enhanced, but the FC between the bilateral prefrontal cortex and the contralesional motor cortex is also significantly enhanced.

While the resting-state FC can provide information about brain function, cortical activity during movement tasks can help us to further explore the interconnections between central and peripheral mechanisms. During multi-joint linkage movement of the affected side, the FC based on the delta HbO_2_ of two channel pairs in the bilateral prefrontal cortex and the motor cortex were significantly stronger than that in the resting state. This phenomenon was not observed for the FC based on delta HbR. This may be because HbR is less sensitive to hemodynamics and less statistically potent than HbO_2_ [35]. In addition, compared with the control group, the FC between the contralesional prefrontal cortex and the ipsilesional motor cortex also increased significantly during multi-joint linkage movement of the affected upper limb in the patients. Evidence suggests that the prefrontal cortex plays a critical role in the speed of processing a movement task and planning movement [36,37,38]. The speed of upper limb movement on the affected side in stroke patients is significantly slower than that of the unaffected side and the control group. Therefore, when completing the same task, the prefrontal cortex needs to consume a large amount of energy, and the FC is correspondingly enhanced. Second, because of damage to the affected brain region, the unilateral multi-joint linkage movement of the upper limb requires the bilateral motor cortex to establish new connections to complete the corresponding motor task through the compensatory action of the prefrontal cortex. In addition, previous studies have indicated that when patients performed complex tasks on the affected side, the brain was activated extensively in the whole brain, especially in the prefrontal cortex [39]; however, when the control group performed the same tasks on the nondominant side, prefrontal cortex activation was small and focused [40]. Overactivation of the prefrontal cortex implies functional compensation. All the above results prove that the prefrontal cortex is involved in compensatory movement in the movement of stroke patients, forming reorganization of the brain network. Moreover, it is also related to the compensatory phenomenon of the limbs during multi-joint linkage movement of the upper limb on the affected side. The recovery of the upper limb’s motor function begins from the shoulder joint, then the elbow, wrist and knuckle joints recover in turn. As a result, the patients moved their hands through the shoulder joint or upper body when performing the multi-joint upper limb task, which also resulted in a stronger FC than that of the healthy controls.

In addition, stroke not only affects the movement of the affected upper limb but also had a certain impact on the movement pattern of the unaffected limb [41]. The FC based on delta HbO_2_ between the contralesional and ipsilesional motor cortex, as well as within the motor cortex, was significantly lower than that in the resting state during multi-joint linkage movement of the unaffected upper limb in stroke patients. This may be due to excessive reliance on the unaffected side of the body in daily life after the affected side of the body has been damaged. As a result, during multi-joint linkage movement the unaffected upper limb, the ipsilesional brain cannot be mobilized to participate in the exercise, thus consuming less energy. In addition, the FC of the ipsilesional ROIs decreased significantly compared with the resting state during movement of the unaffected upper limb by the patients, and only part of the FC of the contralesional side of the patients was greater. That is, in the control group, the unilateral multi-joint linkage movement of the upper limb induced an enhanced FC in the bilateral motor cortex, while in the stroke patients, only part of the contralesional motor cortex was in a state of synchronization, while the synchronization of the ipsilesional motor cortex decreased significantly. This result indicates that not only is the affected side movement affected in stroke patients but also the FC pattern of the whole brain during the movement of the unaffected side is different from that of the healthy group; that is, the synchronization between various brain regions is reduced.

In addition, the motor dysfunction of stroke patients caused by damage to the cerebral cortex measured by only the FMA-UE cannot give clear information about brain reorganization. Imaging methods such as fMRI also have radiation and cannot be monitored during movement tasks. As a noninvasive detection method, fNIRS has important value in clinical applications [42,43]. Therefore, the combination of the hemodynamic response of FMA-UE–related multi-joint linkage tasks of the upper limb measured by fNIRS and the FMA-UE score can more accurately evaluate the recovery of the upper limbs’ motor function in stroke patients. Changes in the FC of the brain provide a theoretical basis for timely adjustment of personalized rehabilitation programs.

However, there were still limitations in this experiment. Although HbO_2_ and HbR were used to jointly calculate the FC to ensure the accuracy of the results, the lack of short-channel regression may be the deficiency of this study. Future studies will consider using short channels to make sure the signal is more accurate.

## 5. Conclusions

In this study, Pearson correlation coefficients were used to describe the channel-wise and ROI-wise FC during specific movement tasks in the control group and the stroke patient group to explore the functional reorganization of the brain in stroke patients in the resting state and during multi-joint linkage movement of the upper limb on the unaffected and affected sides. We found three different compensatory mechanisms established in stroke patients. In the resting state, compensatory connections were established between the ipsilesional prefrontal cortex and the bilateral motor cortex to maintain body balance and stability. During multi-joint linkage movement of the unaffected upper limb, the contralesional motor cortex compensates for part of the function of the ipsilesional side. During multi-joint linkage movement of the affected upper limb, the enhancement of the FC between the contralesional prefrontal cortex and the ipsilesional motor cortex suggests the establishment of compensation. In addition, the FC strength of IPFC-CSMA in the resting state and CSMA-CM1 in the movement of the unaffected upper limb may be a new indicator for evaluating motor function. We also found that the brain abnormalities of stroke patients during the multi-joint linkage movement of the unaffected upper limb can provide theoretical support for the remodeling of brain function and guidance for rehabilitation training.

## Figures and Tables

**Table 1:** The clinical characteristics of the stroke patients.

Title	Age	Sex	Type of Stroke	Hemiparesis Side	FMA-UE	NIHSS	MMSE
Pt 1	42	M	I	L	19	1	29
Pt 2	63	F	I	R	65	2	26
Pt 3	69	M	I	R	63	3	24
Pt 4	42	M	I	L	62	2	29
Pt 5	38	M	I	L	40	2	28
Pt 6	46	M	I	L	9	6	30
Pt 7	62	F	H	L	36	2	23
Pt 8	47	M	H	L	8	9	28
Pt 9	55	M	H	L	29	4	29
Pt 10	51	M	I	R	51	1	28
Pt 11	75	M	I	R	27	5	25
Pt 12	56	M	H	R	65	1	29
Pt 13	57	M	I	R	50	3	27
Pt 14	68	F	H	R	8	10	26
Pt 15	45	M	I	L	42	0	28

Pt, patient; M, male; F, female; H, hemorrhagic stroke; I, ischemic stroke; L, left; R, right; FMA-UE, Fugl–Meyer Assessment of the upper extremity; NIHSS, National Institutes of Health Stroke Scale; MMSE, Mini-Mental State Examination.

## Data Availability

The data presented in this study are available on request from the corresponding author. The data are not publicly available due to restrictions of privacy and ethics.
